# The Use of Vacuum Plasma Surface Treatment to Improve the Hydrophilicity and Wettability of Bone Graft Substitutes and Resorbable Membranes: An In Vitro Study

**DOI:** 10.3390/dj13040141

**Published:** 2025-03-25

**Authors:** Marco Tallarico, Silvio Mario Meloni, Michele Troia, Carlotta Cacciò, Aurea Immacolata Lumbau, Ieva Gendviliene, Francesco Mattia Ceruso, Milena Pisano

**Affiliations:** 1 Medical Surgical and Pharmacy Department, University of Sassari, 07100 Sassari, Italy; mtallarico@uniss.it (M.T.); alumbau@uniss.it (A.I.L.); 2 School of Dentistry, University of Sassari, 07100 Sassari, Italy; michitroia10@gmail.com (M.T.); carlotta.caccio@gmail.com (C.C.); milenapisano@yahoo.it (M.P.); 3 Faculty of Medicine, Vilnus University, 01513 Vilniaus, Lithuania; ieva.gendviliene@gmail.com; 4 San Pietro Fatebenefratelli Hospital, 00189 Rome, Italy; f.m.ceruso@gmail.com

**Keywords:** plasma activation, vacuum plasma surface treatment, guided bone regeneration, socket preservation, biomaterials

## Abstract

**Background/Objectives:** We wished to evaluate in vitro whether vacuum plasma surface treatment of bone graft substitutes and resorbable membranes could improve the hydrophilicity and wettability of the tested materials. **Methods:** A total of 28 sterilized samples were considered for this research and divided into three groups. Six samples were used for the SEM-EDS analysis. The other 22 samples were randomly assigned into the test (plasma-treated, *n* = 11) and control (no treatment, *n* = 11) groups. Vacuum plasma surface treatment was performed in the test group before the SEM-EDS analysis using the ACTILINK reborn with a material holder (Plasmapp Co., Ltd., Daejeon, Republic of Korea). Plasmatreat (Plasmatreat, Steinhagen, Germany) inks were used to evaluate the differences in the hydrophilicity between the test and control groups. The outcome measures were the absorption time, wettability grade, and grade of decontamination after different time cycles. **Results:** After the vacuum plasma surface treatment, the absorption time of the inks statistically decreased in all of the subgroups (*p* < 0.05), while the wettability grade increased. The SEM-EDS analyses showed an increased reduction rate of carbon impurities after up to three vacuum plasma surface treatment cycles. Furthermore, the SEM-EDS analysis did not reveal any areas of damage caused by the multiple treatments. **Conclusions:** Within the limitations of this in vitro study, the vacuum plasma surface treatment increased the hydrophilicity and wettability of the tested biomaterials. Particle bone graft and bone blocks should be treated using longer time programs. Further well-conducted randomized clinical trials with sample size calculations are needed to confirm these preliminary results.

## 1. Introduction

Oral implantology is recognized as a safe and predictable clinical methodology able to ensure long-term results in the field of oral rehabilitation [1]. The osseointegration of dental implants finds its origin in the early 1950s, when Prof. Per-Ingvar Brånemark, a Swedish orthopedic surgeon, originally performed orthopedic experiments on rabbit legs [2]. Currently, an implant is considered osseointegrated when there is no progressive relative movement between the implant and the bone with which it should have direct contact [3]. In the past, one of the most important aspects in achieving osseointegration was the primary stability during the implant placement [4]. From its origins to today, the literature has been focused on purely biologically oriented principles. In relation to the latter, numerous surface treatment methods have been investigated and implemented to enhance the biological surface characteristics of the implants, including implant surface roughness modifications and hydrophilicity improvements within the osseointegration process. According to recent studies, both treatments seem to improve the osseointegration mechanism, with stronger and faster bone formation, allowing for faster osseointegration and successful long-term results [5,6,7,8,9].

Several studies have evaluated the surface treatment of dental implants and abutments [10,11]. Activation of a titanium implant’s surface through plasma treatment could represent a positive strategy for removing contaminants from dental abutments and minimizing peri-implant bone resorption [10], as well as increasing the percentages of new bone in close contact with the implant’s surface [11]. This phenomenon was shown to be mediated in vitro by the increasing protein adsorption and osteoblast adhesion on the titanium surface [12,13,14,15]. However, to the best of the authors’ knowledge and at the time of writing, no research has yet evaluated any treatments that are able to increase the energy surface of bone substitutes and membranes. In an animal study, Ho Jik Yang et al. [12] evaluated the effect of vacuum plasma surface treatment on a human acellular dermal matrix, highlighting the potential effect of the treatment on improving reconstructive surgery outcomes [12]. Vacuum plasma surface treatment has also demonstrated an improvement in cell adhesion, modifying the wettability of the titanium plate’s surface [13,14,15], with a reduction in the contact angle between biological fluids and the implant’s surface that favors the diffusion of osteoblastic cells and leaves no residue after treatment. Some changes in the physicochemical characteristics have been reported, such as the surface free energy, hydrocarbon content, and functional hydroxyl groups, that could potentially influence the inflammatory response in the peri-implant tissue [16]. In a clinical randomized controlled trial, argon plasma treatment demonstrated a reduction in peri-implant bone remodeling, with statistically stronger results at up to 5 years of follow-up [17].

The primary aim of this in vitro study was to evaluate whether vacuum plasma surface treatment of bone graft substitutes and resorbable membranes, commonly used for socket preservation and GBR procedures, could improve the surface energy (hydrophilicity) and wettability of the tested materials. The null hypothesis was that the vacuum plasma surface treatment had no effect on the absorption time or the wettability. A secondary aim was to evaluate, using SEM-EDS analyses, the grade of decontamination after different time cycles. This study was reported according to the CRIS guidelines (Checklist for Reporting In-vitro Studies) [18].

## 2. Materials and Methods

### 2.1. Samples

A total of 28 sterilized samples were considered in this in vitro randomized (test and control) research. No sample size calculation was performed due to no other studies in the scientific literature having compared biomaterials with and without vacuum plasma surface treatment. The maximum number of samples was used according to the availability of the department. The samples included different biomaterials commonly used during socket preservation and/or GBR procedures and are reported as follows:

Fourteen RE-BONE blocks of 10 × 10 × 10 (8) and 10 × 10 × 20 (6) mm (UBGEN SRL, Vigonza, Italy);

Four HEART pericardium membranes (Bioteck SPA, Arcugnano, Italy);

Two cancellous granules, 0.5 g~1 cc, 0.25–1 mm, OSTEOXENON (Bioteck SPA, Italy);

Four cancellous granules, 0.5 g, 0.25–1 mm (non-collagen), BIO-GEN (Bioteck SPA, Italy);

Four XC collagen Xenomatrixes (Bioteck SPA).

A total of 6 out of 28 samples (RE-BONE blocks [UBGEN SRL, Vigonza, Italy]) were used for the SEM-EDS analysis. The other 22 samples were randomly divided into two equal groups of 11 samples (test, plasma-treated, and control, no treatment) and tested to evaluate the absorption times and wettability grades. All of the measurements were performed at the Department of Medicine, Surgery, and Pharmacy, the University of Sassari, Italy. The SEM-EDS analysis of three of the plasma-treated RE-BONE blocks (UBGEN SRL) was performed at the Plasmapp R&D Center (Plasmapp Co, Republic of Korea).

The ACTILINK reborn with a material holder (Plasmapp Co., Ltd., Daejeon, the Republic of Korea) was used to treat the samples. Plasmatreat (Plasmatreat, Steinhagen, Germany) inks with different surface tensions were used. The surface energy of different sterile biomaterials with (test) and without (control) vacuum plasma surface treatment was evaluated by measuring the contact angle (to evaluate the wettability) and the absorption time (to evaluate the hydrophilicity) of the used inks.

### 2.2. Vacuum Plasma Surface Treatment

**Figure 1:**
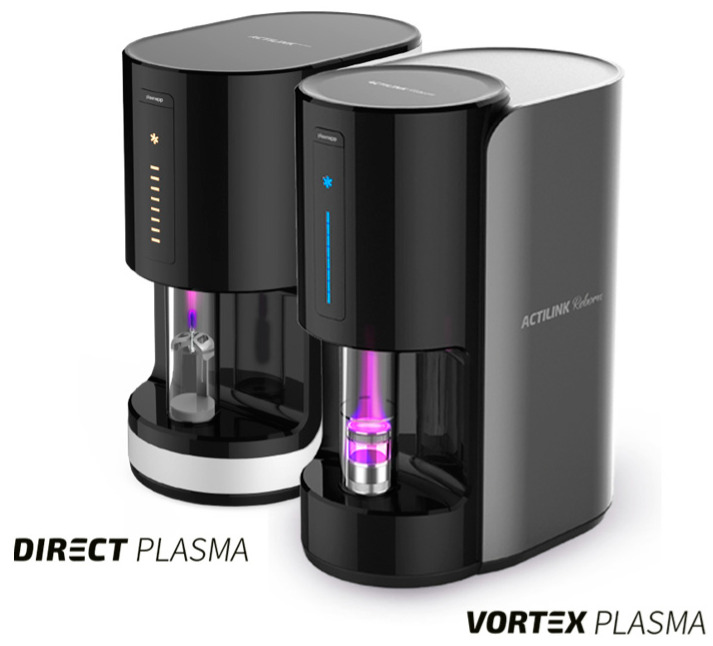
The ACTILINK reborn machine with the vortex holder during vacuum plasma surface treatment on a RE-BONE block.

In the test group, the vacuum plasma surface treatment was performed using the ACTILINK reborn machine (Figure 1) with a customized holder (a vortex holder), designed for easy usage. According to the manufacturers’ protocol, the cycle time of the vacuum plasma surface treatment, named VORTEX PLASMA mode, was 30 s. All of the biomaterials in the test group underwent the same vacuum plasma surface treatment cycle time. On the contrary, none of the biomaterials in the control group received any type of treatment. In the test group, after opening the sterile box, the tested biomaterial was taken with a sterile tweezer and inserted into the sterile vortex holder, and finally, it was placed into the ACTILINK machine for the vacuum plasma surface treatment. Once the treatment ended, both the treated and untreated biomaterials were inserted using a sterile tweezer into the sterilized holder, and five drops of the Plasmatreat inks, with two different surface tensions (56 and 72 mN/m), were dropped onto each sample. Immediately after, the respective absorption time and wettability grade were recorded, photographed, and critically compared to evaluate the hydrophilicity and the contact angle between the ink and the surface of the used biomaterials. Surface tensions of 56 and 72 mN/m were used because the value of 56 mN/m was closest to that of human blood.

### 2.3. SEM Analysis

In order to evaluate the effect of the vacuum plasma treatment time on the decontamination (reducing the carbon impurities) of a bone block surface, three RE-BONE blocks (UBGEN SRL) were analyzed using the SEM-EDS system after up to three vacuum plasma surface treatment cycle times. Six RE-BONE blocks of 10 × 10 × 20 mm (UBGEN SRL) were used for the SEM-EDS examination. Two RE-BONE blocks (UBGEN SRL) were treated under three different cycles each ([A] 30 s, [B] 60 s, and [C] 90 s) before the vacuum plasma surface treatment. The ACTILINK reborn machine was used with the VORTEX PLASMA mode. After every treatment cycle, the bone blocks were analyzed under a scanning electron microscope (SEM, Thermo Fisher Scientific, Phenom XL, Waltham, MA, USA) connected to an energy-dispersive X-ray spectroscope (EDS) to allow for a targeted analysis of the samples’ surfaces.

### 2.4. Outcome Measures

Absorption time and wettability grade were evaluated from videos recorded during the procedures (Blackmagic Design Pocket Cinema Camera 4K, Blackmagic, Fremont, CA, USA). The test and control groups were tested in comparison at the same time. Two researchers performed all of the tests (M.T. and M.T).

**Figure 2:**
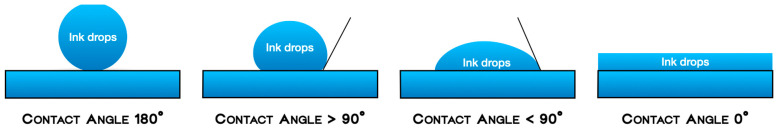
The ACTILINK reborn machine with the vortex holder during vacuum plasma surface treatment of a RE-BONE block.

- Absorption time was defined as the interval, in seconds, from the moment that the last drop touched the biomaterial’s surface to the moment when all of the ink drops had been absorbed into the biomaterial. The recorded video was evaluated using a video editing application (iMovie for MacOS), and the absorption time was measured using the expanded timeline. All of the measurements were repeated three times by two different operators (M.T. and M.T.). The mean value and standard deviation (SD) were calculated.
- Wettability (generally referred to as hydrophilicity) was defined as the spreads of the ink drops over the biomaterials’ surfaces, measured by the flatness of a droplet on the solid surface. The four grades of wettability were defined as follows:

Null grade: The ink drops remained in the same position at which they were dropped with a contact angle of 180°; Low grade: When the ink stain slightly widened on the surface, with a contact angle > 90°; Medium grade: When moderate expansion of the ink stain on the surface was appreciable and the contact angle was <90°; High grade: When the ink stain had definitely been absorbed, with a contact angle of 0° (Figure 2).

- SEM analysis. Scanning electron microscopy was employed to visualize high-resolution images of the sample surfaces. Using the SEM, the surface topography in the images was analyzed. In particular, the SEM images were used to evaluate the reduction rate of carbon impurities with three different cycle times. The energy-dispersive X-ray spectroscope (EDS) detector was used to measure the energy of the emitted photons in the X-ray electromagnetic spectrum and to obtain chemical information (the atomic percentage).

### 2.5. Statistical Analysis

The entire data analysis was carried out according to a pre-established analysis plan. A biostatistician with expertise in dentistry analyzed the data using Ky Plot 2.0 software, Informer Technologies, Inc. NY, USA, without knowing the group codes. The mean values and standard deviations were calculated for each measurement. A two-sample Kolmogorov–Smirnov test calculator was used to compare the absorption time between groups. Statistical comparisons were conducted at the 0.05 level of significance.

## 3. Results

**Figure 3:**
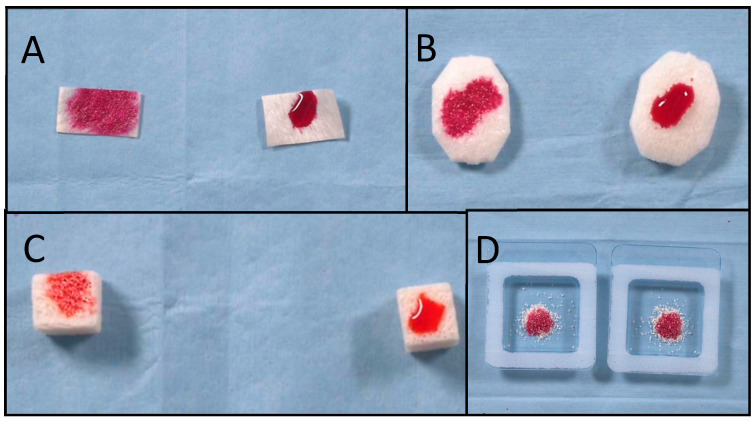
(**A**) Pericardium membranes, (**B**) collagen Xenomatrixes, (**C**) RE-BONE blocks, and (**D**) collagen bone grafts. In all boxes, the plasma-treated biomaterial is on the left, and the untreated biomaterial is on the right.

A total of 28 samples were used. The absorption time and wettability of 22 samples after various inks with different surface tensions had been dropped onto them were evaluated in both groups. The vacuum plasma surface treatment statistically reduced the absorption time in all of the treated samples (*p* value < 0.05, Table 1). Moreover, for all of the samples except for the OSTEOXENON cancellous granules with 56 mN/m ink, the wettability in the test group was higher-grade compared to that in the control group. The highest difference in wettability was found for the pericardium membrane, with a high grade in the test group compared with a null grade in the control group. The best outcomes were found for vacuum plasma surface treatment of the bone blocks, collagen membranes, pericardium membranes, and collagen bone grafts, respectively. All of the data are reported in Table 1. Explanatory pictures are reported in Figure 3.

**Figure 4:**
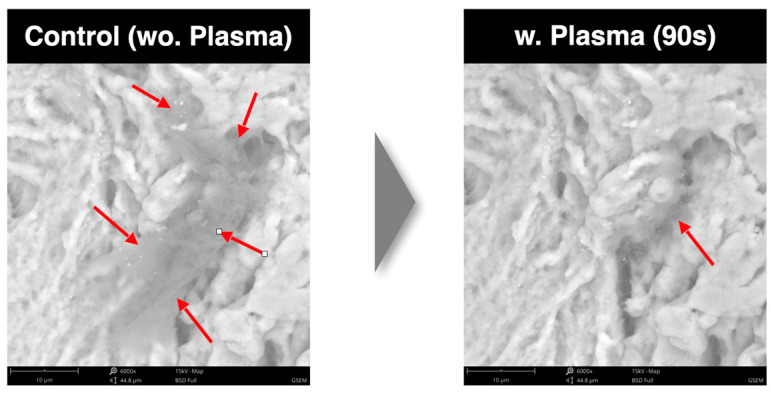
SEM analysis showing percentage reduction rate of carbon impurities for control and test groups after three vacuum plasma surface treatments. Legend: wo = without; w = with.

**Figure 5:**
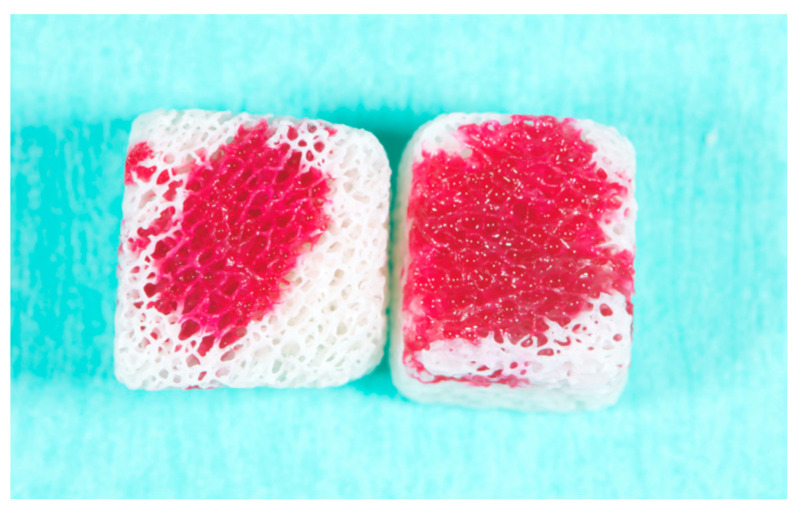
Top view of a sample. Great wettability after three time cycles (right sample) of vacuum plasma surface treatment.

Six bone blocks (three after vacuum plasma surface treatments and three without any treatment) were evaluated in the SEM-EDS analysis following one, two, and three time cycles, respectively. The reduction rate of carbon impurities tended to increase after three time cycles compared to that under one and two time cycles. Furthermore, the SEM-EDS analysis showed no damage to the biomaterials after multiple (up to three times, 90 s, Figure 4) vacuum plasma surface treatments, while the top of the surface showed better wettability after three time cycles (Figure 5). All of these data are reported in Table 2 and Table 3.

## 4. Discussion

This study aimed to evaluate whether the effect of vacuum plasma surface treatment on various biomaterials used for socket preservation and GBR procedures could improve their hydrophilicity and wettability. The hydrophilicity was measured through the ink absorption time and the wettability grade. The ink absorption time of the plasma-treated samples was significantly shorter than that of the untreated samples. Hence, the null hypothesis of no difference was partially rejected. These initial in vitro tests indicated that vacuum plasma surface treatment had a positive effect on the bone blocks, pericardium membranes, collagen matrices, and collagen bone grafts in terms of both the absorption time and wettability grade. On the contrary, vacuum plasma surface treatment failed to reduce the absorption time for non-collagen bone grafts. This indicates that the vacuum plasma surface treatment converted the dried biomaterials’ surfaces from hydrophobic to highly hydrophilic surfaces. However, the initial surface characteristics are important in defining the expected outcomes.

Increasing the overall treatment time, up to three time cycles, improved the outcomes. On possible explanation is that the carbon impurities tend to decrease after three time cycles [12]. In vivo, vacuum plasma surface treatment of a human acellular dermal matrix showed enhanced fibroblast infiltration, indicating improved biocompatibility [12]. In the present research, the vacuum plasma surface treatment exhibited some positive effects in terms of decontamination of the treated biomaterials and in terms of activation of the surfaces, reducing the impurities and boosting their hydrophilicity.

Several studies in the literature have confirmed the effectiveness of plasma treatments in terms of cell adhesion and fibroblast activity. However, the main topics of these studies were dental abutments and implant surfaces [19,20,21,22].

Surface wettability is one of the most important parameters affecting the biological response to an implanted material, affecting protein adsorption, platelet adhesion/activation, blood coagulation, and cell and bacterial adhesion. In the present research, inks were used to measure the absorption time and wettability grade of the tested biomaterials. The surface tension of blood plays an important role in the human body [23]. According to Hrncír and Rosina [24], the surface tension of blood, assessed in a group of 71 healthy subjects using the drop method at a temperature of 22 degrees Celsius, was 55.89 × 10^(−3)^ N × m^(−1)^, with an SD = 3.57 × 10^(−3)^ N × m^(−1)^. Considering that changes in the surface tension behavior of human biological fluid are characteristic in some diseases [24], for the present research, inks with 56 mN/m and 72 mN/m surface tensions were used.

Analyzing the data collected in the present research, it was found that in the control group, the ink with a higher surface tension (72 mN/m) was absorbed quicker than the ink with a lower surface tension (56 mN/m) by both the bone blocks and collagen Xenomatrixes but not by the non-collagen bone grafts. On the contrary, in the test group, the difference was not relevant. These results may mean that after vacuum plasma surface treatment, variations in surface tension could be less relevant to the healing process.

After up to three time cycles, the SEM imaging of the surface topography revealed no changes under the vacuum plasma surface treatments, and no physical damage was observed. The increase in the hydrophilicity, the reduction in the impurity grade, and the preservation of the original structure, without any physical damage to the plasma-treated bone blocks, should be considered evidence of the improved biocompatibility and potentially biointegration of the tested materials.

Vacuum plasma treatment is widely used in medicine to improve biocompatibility and biointegration in reconstructive surgeries [12]. Findings from a similar in vitro research highlight the potential of plasma treatment to enhance the performance of hADMs in clinical settings, offering a promising avenue for improving reconstructive surgery outcomes [25]. In addition, vacuum plasma treatment is also used in other fields, such as to increase the electrical properties of organosilicate films or the wettability of polyetheretherketone (PEEK) polymers [26].

The main limitation of the present research is the small number of samples and, of course, its in vitro nature. Another limitation is that the wettability contact angle should have been provided instead of the wettability grade level. Even if the results from the present research are encouraging for vacuum plasma surface treatment, in vitro data do not allow us to draw any definitive clinical conclusions. However, analyzing the data from the SEM-EDS analysis revealed that the carbon impurity rate on the bone blocks’ surfaces tended to decrease when increasing the number of time cycles; therefore, increasing the time cycles up to three could be suggested depending on the bone graft substitutes used. Moreover, the absorption time and wettability grade also improved after two and three time cycles. In relation to the latter, pericardium membranes and bone grafts, particularly non-collagen bone grafts, could be treated by increasing the number of cycles to two or three to reduce the degree of carbon impurities and increase their hydrophilicity. However, further clinical randomized controlled trials are needed to confirm these preliminary results. Another limitation of this research is that since there were not many similar studies in the scientific literature, it was not easy to define a criterion for interpreting the results, especially in regard to the wettability grade of the tested bone graft substitutes and resorbable membranes. Data from similar in vitro and in vivo studies have confirmed the positive effect of plasma treatment on implants and abutments when using argon plasma [12,13,14,15,19]. Regarding the latter, the data from this in vitro research must be considered a preliminary report to encourage further clinical evaluations.

## 5. Conclusions

The vacuum plasma surface treatment statistically increased the hydrophilicity of most of the tested biomaterials, reducing the absorption time and increasing the wettability grade. In addition, the rate of carbon impurities could be reduced by increasing the cycle time. However, further randomized controlled studies with sample size calculations are needed to confirm these preliminary results.

## Figures and Tables

**Table 1:** Absorption time and wettability between test and control groups.

	Test (Plasma)		Control (No-Treatment)		
	Absorption Time ^2^	Wettability Grade	Absorption Time	Wettability Grade	*p* Value ^3^
RE-BONE Blocks 10 × 10 × 20 mm (*n* = 2) 56 mN/m ink ^1^	0.19 ± 0.03 s	Medium	23.27 ± 0.10 s	Low	0.0048
RE-BONE Blocks 10 × 10 × 10 mm (*n* = 6) 72 mN/m ink ^1^	0.3 ± 0.02 s	Medium	7.37 ± 0.06 s	Low	0.0048
HEART Pericardium Membranes (*n* = 4) 56 mN/m ink ^1^	15.37 ± 0.16 s	High	∼30 min	Null	0.0048
Cancellous Granules, OSTEOXENON (*n* = 2) 56 mN/m ink ^1^	1.72 ± 0.10 s	Medium	3.19 ± 0.12 s	Medium	0.0043
Cancellous Granules, BIO-GEN (*n* = 2) 56 mN/m ink ^1^	2.36 ± 0.18 s	High	1.19 ± 0.05 s	Medium	0.0048
Cancellous Granules, BIO-GEN (*n* = 2) 72 mN/m ink ^1^	2.72 ± 0.10 s	High	1.90 ± 0.04 s	Medium	0.0047
XC Collagen Xenomatrix (*n* = 2) 56 mN/m ink ^1^	3.88 ± 0.14 s	Medium	∼30 min	Null	0.0048
XC Collagen Xenomatrix (*n* = 2) 72 mN/m ink ^1^	3.80 ± 0.14 s	High	13.49 ± 0.18 s	Medium	0.0048

Surface tension ^1^; absorption time measured in seconds ± standard deviation (SD) ^2^. Comparison between absorption time values ^3^.

**Table 2:** EDS analysis between test and control groups showing atomic percentage of carbon impurities (at) between test and control groups and reduction rate.

	Plasma 30 s			Plasma 90 s		
	No Plasma (at.%)	Plasma (at.%)	Reduction Rate (%)	No Plasma (at.%)	Plasma (at.%)	Reduction Rate (%)
1.	74.98	70.29	4.69	42.17	20.92	50.39
2.	70.12	62.93	10.25	24.73	24.05	2.75
3.	61.99	59.34	4.27	84.68	47.30	44.14
Avg.	69.03	64.2	6.4	50.5	30.8	32.4

**Table 3:** Absorption time and wettability after different time cycles.

	Test (Plasma) 30′		Test (Plasma) 60′		Test (Plasma) 90′	
	Absorption Time ^2^	Wettability Grade	Absorption Time ^2^	Wettability Grade	Absorption Time ^2^	Wettability Grade
RE-BONE Blocks 10 × 10 × 20 mm 56 mN/m ink ^1^	0.19 ± 0.03 s (*n* = 20)	Medium	0.08 ± 0.02 s (*n* = 20)	High	0.07 ± 0.04 s (*n* = 20)	High

Surface tension ^1^; absorption time measured in seconds ± standard deviation (SD) ^2^.

## Data Availability

The data can be made available on request to the authors.

## References

[1] Busenlechner D., Fürhauser R., Haas R., Watzek G., Mailath G., Pommer B. (2014). Long-term implant success at the Academy for Oral Implantology: 8-year follow-up and risk factor analysis. J. Periodontal Implant. Sci..

[2] Kim T.-I. (2014). A Tribute to Dr. Per-Ingvar Brånemark. J. Periodontal Implant. Sci..

[3] Mavrogenis A.F., Dimitriou R., Parvizi J., Babis G.C. (2009). Biology of Implant Osseointegration. J. Musculoskelet. Neuronal Interact..

[4] Peñarrocha D.M., Cavani U., Cuadrado L. (2019). Atlas of Immediate Dental Implant Loading.

[5] Wong M., Eulenberger J., Schenk R., Hunziker E. (1995). Effect of surface topology on the osseointegration of implant materials in trabecular bone. J. Biomed. Mater. Res..

[6] Wennerberg A., Albrektsson T. (2010). On implant surfaces: A review of current knowledge and opinions. Int. J. Oral Maxillofac. Implant..

[7] Wennerberg A., Albrektsson T. (2009). Effects of titanium surface topography on bone integration: A systematic review. Clin. Oral Implant. Res..

[8] Beutner R., Michael J., Schwenzer B., Scharnweber D. (2010). Biological nano-functionalization of titanium-based biomaterial surfaces: A flexible toolbox. J. R. Soc. Interface.

[9] Sun X.D., Liu T.T., Wang Q.Q., Zhang J., Cao M.S. (2023). Surface Modification and Functionalities for Titanium Dental Implants. ACS Biomater. Sci. Eng..

[10] Canullo L., Tallarico M., Peñarrocha M., Corrente G., Fiorellini J., Peñarrocha D. (2017). Plasma of Argon Cleaning Treatment on Implant Abutments in Periodontally Healthy Patients: Six Years Postloading Results of a Randomized Controlled Trial. Int. J. Periodontics Restor. Dent..

[11] Canullo L., Tallarico M., Botticelli D., Alccayhuaman K.A.A., Neto E.C.M., Xavier S.P. (2018). Hard and Soft Tissue Changes around Implants Activated Using Plasma of Argon: A Histomorphometric Study in Dog. Clin. Oral Implant. Res..

[12] Yang H.J., Lee B., Shin C., You B., Oh H.S., Lee J., Lee J., Oh S.K., Oh S.H. (2024). Improvement in Biocompatibility and Biointegration of Human Acellular Dermal Matrix through Vacuum Plasma Surface Treatment. Bioengineering.

[13] Kawai H., Shibata Y., Miyazaki T. (2004). Glow Discharge Plasma Pretreatment Enhances Osteoclast Differentiation and Survival on Titanium Plates. Biomaterials.

[14] Shibata Y., Hosaka M., Kawai H., Miyazaki T. (2002). Glow Discharge Plasma Treatment of Titanium Plates Enhances Adhesion of Osteoblast-like Cells to the Plates through the Integrin-Mediated Mechanism. Int. J. Oral Maxillofac. Implant..

[15] Canullo L., Genova T., Mandracci P., Mussano F., Abundo R., Fiorellini J.P. (2017). Morphometric Changes Induced by Cold Argon Plasma Treatment on Osteoblasts Grown on Different Dental Implant Surfaces. Int. J. Periodontics Restor. Dent..

[16] Noro A., Kaneko M., Murata I., Yoshinari M. (2013). Influence of Surface Topography and Surface Physicochemistry on Wettability of Zirconia (Tetragonal Zirconia Polycrystal). J. Biomed. Mater. Res. Biomater..

[17] Canullo L., Tallarico M., Peñarrocha-Oltra D., Monje A., Wang H., Peñarrocha-Diago M. (2016). Implant Abutment Cleaning by Plasma of Argon: 5-Year Follow-Up of a Randomized Controlled Trial. J. Periodontol..

[18] Krithikadatta J., Gopikrishna V., Datta M. (2014). CRIS Guidelines (Checklist for Reporting In-vitro Studies): A concept note on the need for standardized guidelines for improving quality and transparency in reporting in-vitro studies in experimental dental research. J. Conserv. Dent..

[19] Canullo L., Genova T., Chinigò G., Iacono R., Pesce P., Menini M., Mussano F. (2024). Vacuum Plasma Treatment Device for Enhancing Fibroblast Activity on Machined and Rough Titanium Surfaces. Dent. J..

[20] Jeon H.J., Jung A., Kim H.J., Seo J.S., Kim J.Y., Yum M.S., Gweon B., Lim Y. (2022). Enhanced Osteoblast Adhesion and Proliferation on Vacuum Plasma-Treated Implant Surface. Appl. Sci..

[21] Pesce P., Menini M., Santori G., Giovanni E.D., Bagnasco F., Canullo L. (2020). Photo and Plasma Activation of Dental Implant Titanium Surfaces. A Systematic Review with Meta-Analysis of Pre-Clinical Studies. J. Clin. Med..

[22] Lee H., Jeon H.J., Jung A., Kim J., Kim J.Y., Lee S.H., Kim H., Yeom M.S., Choe W., Gweon B. (2022). Improvement of osseointegration efficacy of titanium implant through plasma surface treatment. Biomed. Eng. Lett..

[23] Fathi-Azarbayjani A., Jouyban A. (2015). Surface tension in human pathophysiology and its application as a medical diagnostic tool. Bioimpacts.

[24] Hrncír E., Rosina J. (1997). Surface tension of blood. Physiol. Res..

[25] Baklanov M.R., Gismatulin A.A., Naumov S., Perevalov T.V., Gritsenko V.A., Vishnevskiy A.S., Rakhimova T.V., Vorotilov K.A. (2024). Comprehensive Review on the Impact of Chemical Composition, Plasma Treatment, and Vacuum Ultraviolet (VUV) Irradiation on the Electrical Properties of Organosilicate Films. Polymers.

[26] Primc G. (2022). Strategies for Improved Wettability of Polyetheretherketone (PEEK) Polymers by Non-Equilibrium Plasma Treatment. Polymers.

